# Identification and Dynamic Remodeling of Bivalent Chromatin Regions in Sheep Early Embryos and ESCs

**DOI:** 10.3390/ijms27209192

**Published:** 2026-10-16

**Authors:** Linlin Song, Hua Yang, Lu Gan, Haonan Chen, Zengguang Liao, Ershuai Kuang, Mingtian Deng

**Affiliations:** Jiangsu Livestock Embryo Engineering Laboratory, College of Animal Science and Technology, Nanjing Agricultural University, No. 1 Weigang, Nanjing 210095, China

**Keywords:** sheep, embryonic stem cells, bivalent chromatin, H3K4me3, H3K27me3

## Abstract

Bivalent chromatin, marked by H3K4me3 and H3K27me3, contributes to maintaining developmental genes in a poised state. However, it remains unclear whether sheep embryonic stem cells (ESCs) establish bivalent domains. In this study, we identified bivalent chromatin regions and characterized the dynamics of H3K4me3, chromatin accessibility, and expression of bivalent chromatin-associated genes in sheep early embryos and ESCs. Using an overlap threshold of >200 bp between H3K4me3 and H3K27me3 peaks, we identified 40,543 bivalent regions in sheep ESCs. These regions preferentially occurred at promoters and were enriched in RNA processing, translation, and ribonucleoprotein complex biogenesis. By integrating Cleavage Under Targets and Tagmentation (CUT&Tag), Assay for Transposase-Accessible Chromatin using sequencing (ATAC-seq), and RNA-seq data across preimplantation development from MII oocytes to hatching blastocysts, we found that ESC-defined bivalent regions underwent dynamic changes in H3K4me3 enrichment and chromatin accessibility, with distinct patterns across developmental stages. Bivalent-associated genes also exhibited stage-specific expression patterns, and key pluripotency- and lineage-associated transcription factors, including SOX2, SOX17, POU5F1, and ZFP42, showed distinct developmental expression profiles. Moreover, formative ESCs displayed higher transcriptional activity and chromatin accessibility at bivalent regions than that of primed ESCs. Together, these results characterize the genomic distribution of bivalent chromatin in sheep ESCs and its stage-specific remodeling during preimplantation development, providing a resource for further investigation of epigenetic regulation during early embryonic development in sheep.

## 1. Introduction

Embryonic stem cells (ESCs) are pluripotent cells derived from the inner cell mass (ICM) of blastocysts. They possess unlimited self-renewal capacity and can differentiate into all cell types of the three embryonic germ layers, making them an essential in vitro model for investigating early embryonic development [1,2]. ESCs in mice and humans were established in 1981 [3] and 1998 [4], respectively. However, establishing stable ESC lines in livestock species has remained challenging, partly because pluripotency regulatory networks and culture requirements differ by species [5]. In recent years, as culture systems have been optimized, ESCs from pigs [6], cattle [7], and sheep [8] have been successfully generated. In sheep, although the pluripotency and transcriptomic landscapes of ESCs have been characterized [9,10], the regulatory mechanisms governing ESC pluripotency remain poorly understood.

Previous studies show that epigenetic mechanisms tightly control expression of core transcription factors, including Oct4, Sox2, and Nanog [11], thereby maintaining ESC pluripotency [12]. Among these mechanisms, trimethylation of Histone 3 lysine 4 (H3K4me3) and lysine 27 (H3K27me3) plays a central role in coordinating gene expression programs during self-renewal and differentiation [13]. H3K4me3 is predominantly enriched around transcription start sites (TSSs) and is generally associated with transcriptionally active or permissive promoters [14], whereas H3K27me3 is deposited by the Polycomb Repressive Complex 2 (PRC2) and primarily mediates transcriptional repression [15]. These marks are dynamically regulated by their cognate writers and erasers. In ESCs, H3K4me3 is deposited by SETD1/MLL (KMT2) family methyltransferase complexes and removed by KDM5 (JARID1) family demethylases, and the balance between these opposing enzymatic activities is continuously adjusted to coordinate gene expression programs during self-renewal and differentiation [14,16]. In particular, KDM5B (JARID1B), a key member of this family, co-localizes with H3K4me3 at promoters and active enhancers in ESCs, where it safeguards the focal enrichment of the mark at these sites and prevents aberrant spreading into gene bodies [17]. More recently, KDM5B has been shown to erase H3K4me3 during the totipotency-to-pluripotency transition in mouse preimplantation embryos, restricting the mark to active promoters and ensuring proper lineage segregation at the blastocyst stage [18]. Together with the KMT2 writer complexes, KDM5B thus contributes to the precise spatial control of H3K4me3 that is essential for ESC self-renewal and early lineage commitment. H3K27me3, in contrast, is written at Polycomb target genes by the EZH1/EZH2 catalytic subunits of PRC2 and erased by KDM6 family demethylases (UTX/KDM6A and JMJD3/KDM6B), and its dynamic turnover is equally important for proper lineage commitment of ESCs [16,19].

Recently, studies revealed that H3K4me3 and H3K27me3 coexist at the promoters of developmental genes, forming a distinctive chromatin state known as bivalent chromatin in mouse ESCs [20,21]. Bivalent chromatin has been proposed to keep developmental genes in a transcriptionally poised state, potentially allowing their rapid activation or stable repression in response to differentiation cues [20,22]. In mouse and human ESCs, thousands of bivalent promoters have been identified, and they are strongly associated with promoters of developmental regulator genes, most of which contain CpG islands [23,24,25,26,27]. Moreover, the prevalence of bivalent domains varies across pluripotent states, with mouse ESCs cultured in the naive ground state harboring fewer bivalent domains than serum-cultured ESCs [28]. These observations suggest that bivalent chromatin is a dynamic feature of pluripotent cells rather than a fixed chromatin state. Consistent with this view, bivalent chromatin undergoes extensive remodeling during peri-implantation development in mice, and the resolution of a subset of bivalent domains has been implicated in epiblast progression and embryogenesis. These findings suggest that bivalent chromatin is not a static feature of ESCs but is dynamically associated with developmental potential and transitions between pluripotent states. However, it remains unclear whether bivalent domains are established in sheep ESCs. Moreover, because preimplantation development involves extensive chromatin remodeling and the progressive establishment of lineage-specific transcriptional programs, how ESC-associated bivalent regions are remodeled during these processes remains unresolved.

In the present study, we mapped H3K4me3 and H3K27me3 profiles in sheep ESCs and identified bivalent chromatin domains by the co-enrichment of these two modifications. We further characterized the dynamics of H3K4me3 and chromatin accessibility in these bivalent chromatin regions, as well as the expression of bivalent-associated genes during early sheep embryogenesis. We also compared the transcriptional activity and chromatin accessibility at these regions between formative and primed sheep ESCs. Our data delineate the bivalent chromatin landscape of sheep ESCs and provide initial insights into its developmental remodeling and potential regulatory relevance during early embryogenesis.

## 2. Results

### 2.1. Identification and Characterization of Bivalent Chromatin Regions in Sheep ESCs

**Figure 1:**
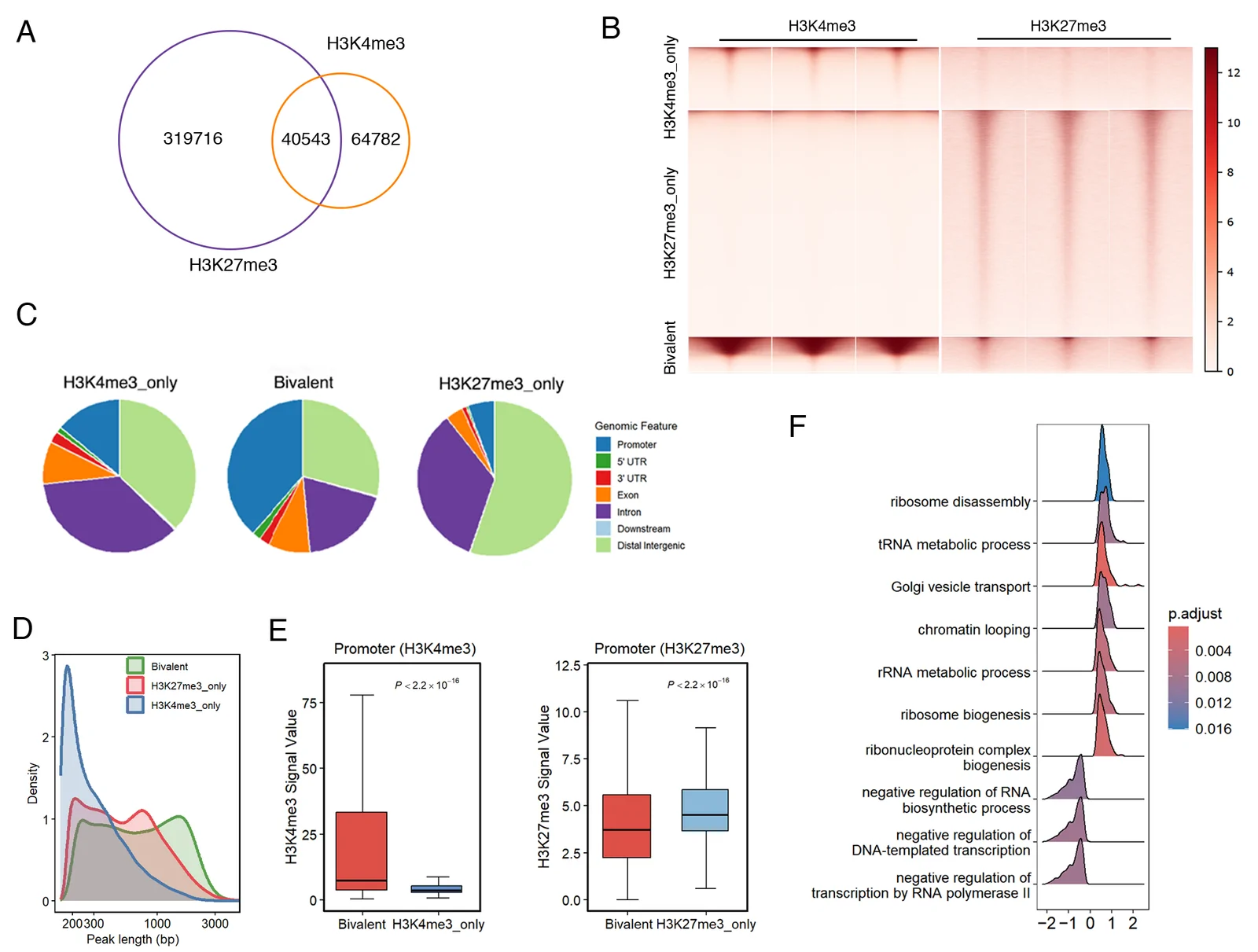
Identification and characterization of bivalent chromatin regions in sheep ESCs. (**A**) Overlap among H3K4me3-only, H3K27me3-only, and bivalent peaks in sheep ESCs. (**B**) Heatmap of normalized H3K4me3 and H3K27me3 signal intensities centered on H3K4me3-only, H3K27me3-only, and bivalent regions. (**C**) Genome-wide distribution frequency in H3K4me3-only, H3K27me3-only, and bivalent regions. (**D**) Density distribution of peak lengths for H3K4me3-only, H3K27me3-only, and bivalent regions. (**E**) Boxplots comparing H3K4me3 and H3K27me3 signal intensities at bivalent (n = 13,924), H3K4me3-only (n = 7067), and H3K27me3-only (n = 11,826) promoters. *p*-values from a two-sided Wilcoxon test; effect sizes (Cliff’s delta) are reported in the main text. (**F**) Over-representation analysis (ORA) of representative Gene Ontology (GO) terms among bivalent-associated genes; enrichment was assessed on the fixed gene set defined by peak overlap.

We first identified H3K4me3 and H3K27me3 bivalent chromatin regions in sheep ESCs. Venn plot and heatmap analyses revealed that 319,716 regions showed only H3K27me3 enrichment, while 64,782 regions showed only H3K4me3 enrichment. Of particular interest, 40,543 regions were occupied by both H3K4me3 and H3K27me3 with more than 200 bp of overlap and were therefore defined as bivalent chromatin regions (Figure 1A,B). To assess the robustness of bivalent-region identification to the operational overlap criterion, sensitivity analyses were performed using alternative absolute overlap thresholds of ≥100 bp and ≥300 bp, which identified 47,779 and 22,404 bivalent regions, respectively; in addition, a relative-overlap criterion requiring >50% of the shorter of the two overlapping peaks to be shared yielded 42,329 bivalent regions. The regions identified using the primary ≥200 bp criterion showed substantial concordance with those identified using the >50% relative-overlap criterion, with 36,388 regions shared between the two sets, corresponding to 89.75% of the ≥200 bp set and 85.96% of the relative-overlap set (Jaccard similarity = 0.892), indicating that bivalent-region identification is largely insensitive to the choice of overlap criterion. Genomic annotation showed that bivalent peaks occurred at promoters, 5′ untranslated regions (UTRs), 3′ UTRs, exons, introns, distal intergenic regions, and immediate downstream regions, with the most pronounced promoter enrichment (38.40%, Figure 1C). Detailed statistics of peak counts and genomic annotation percentages are listed in Table S3. Length-distribution analysis showed that H3K4me3-only peaks were concentrated around 200 bp, H3K27me3-only peaks ranged from 200 bp to 1000 bp, and bivalent peaks spanned from 200 bp to 2000 bp (Figure 1D), indicating that bivalent regions form extended chromatin territories rather than focal promoter marks. Boxplot analysis at promoters revealed that bivalent promoters displayed a substantially higher H3K4me3 signal than H3K4me3-only promoters (median difference = 29.095, two-sided Wilcoxon rank-sum test, *p* < 2.2 × 10^−16^, Cliff’s delta = 0.503, large effect). In contrast, bivalent promoters exhibited a lower H3K27me3 signal relative to H3K27me3-only promoters (median difference = −1.363, *p* < 2.2 × 10^−16^, Cliff’s delta = −0.412, medium effect; Figure 1E). Together, these results indicate that the two marks coexist at bivalent promoters without mutual attenuation and that the H3K4me3 signal is even amplified in the bivalent state. GO over-representation analysis (ORA) revealed that genes associated with bivalent peaks were significantly enriched in biological processes related to ribosome disassembly, the tRNA metabolic process, Golgi vesicle transport, and chromatin looping (Figure 1F), suggesting that bivalent-associated genes in sheep ESCs encompass both developmental regulators and genes involved in fundamental cellular processes.

### 2.2. Dynamic Changes in H3K4me3 at Bivalent Chromatin Regions During Sheep Early Embryonic Development

**Figure 2:**
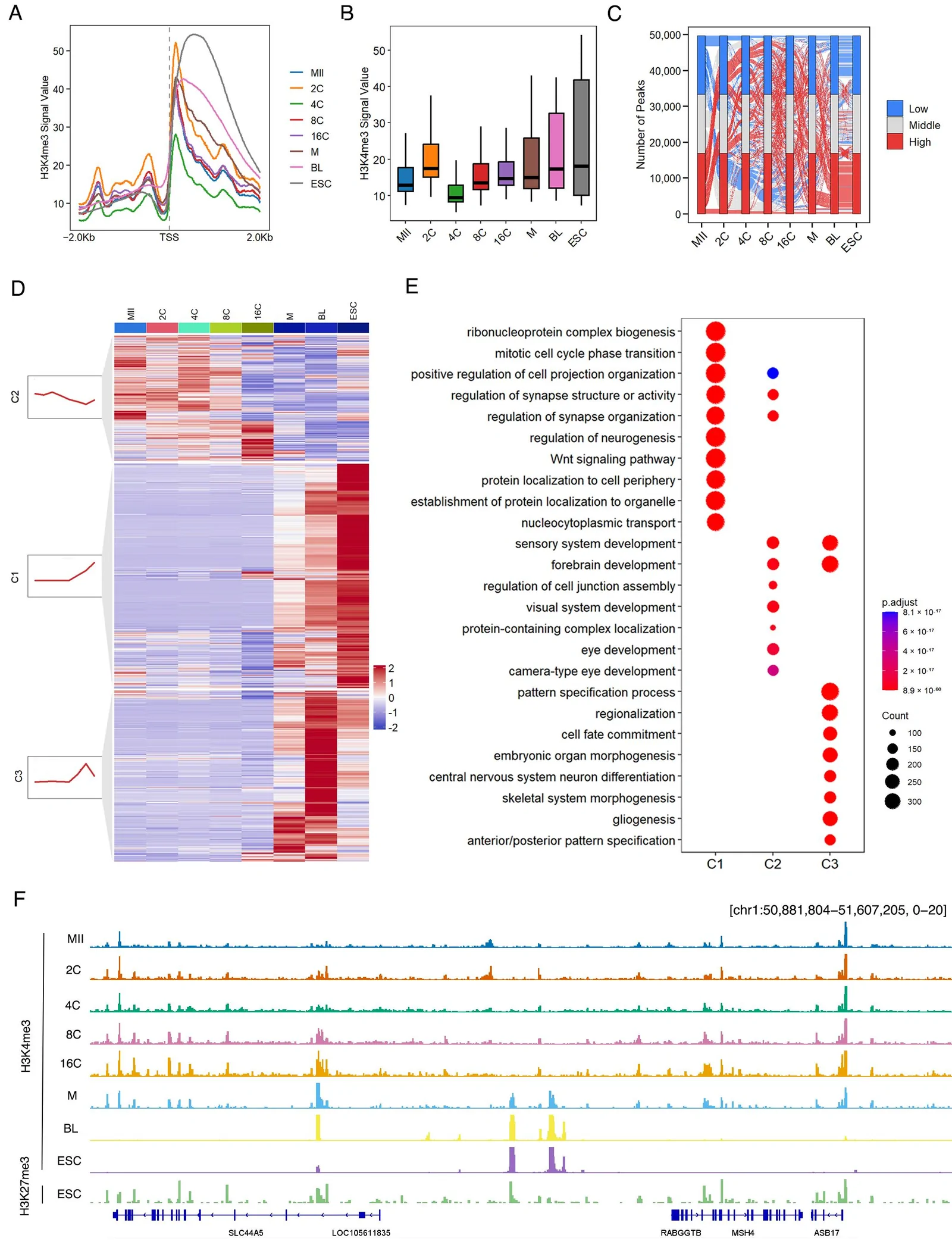
Dynamic changes in H3K4me3 at bivalent chromatin regions during sheep early embryonic development. (**A**) Metaplot of H3K4me3 mean coverage at transcription start sites (TSSs) across MII oocytes, 2-, 4-, 8-, and 16-cell stage embryos, morula, blastocysts, and ESCs. (**B**) Boxplot of genome-wide H3K4me3 signal intensity across the indicated developmental stages. (**C**) Stacked plot showing the numbers of H3K4me3 peaks with low, middle, and high signal intensities at each stage. (**D**) Heatmap and k-means clustering (k = 3) of H3K4me3 signals across developmental stages, with cluster sizes indicated. (**E**) Gene Ontology analysis of genes associated with each H3K4me3 cluster. (**F**) An IGV snapshot showing H3K4me3 distribution in sheep oocytes and the early embryos, together with H3K4me3 and H3K27me3 in ESCs, at the chromosome 3 region.

We next traced the dynamics of the H3K4me3 signal across early embryonic development at bivalent chromatin regions. Metaplot and boxplot analyses showed that the H3K4me3 signal is lowest at the 4C stage and rises progressively thereafter, reaching its maximum at the ESC stage (Figure 2A,B). The Sankey plot revealed H3K4me3 dynamics in specific regions during early embryonic development (Figure 2C). The H3K4me3 signal at bivalent regions was further classified into three clusters by the k-means algorithm (Figure 2D). In cluster 1 (C1, n = 12,027), the H3K4me3 signal was comparatively low through early and mid-preimplantation and increased at the blastocyst but decreased at the ESC, and genes in C1 were enriched for ribonucleoprotein complex biogenesis and nucleocytoplasmic transport. In cluster 2 (C2, n = 16,290), H3K4me3 displayed a relatively high signal at early stages followed by a gradual decline after the 16-cell stage and was enriched for genes involved in positive regulation of cell projection organization and regulation of synapse organization. In cluster 3 (C3, n = 21,407), H3K4me3 exhibited a low signal at early stages but strong upregulation at the blastocyst and ESC stages and was enriched for genes involved in cell fate commitment and embryonic organ morphogenesis (Figure 2D,E). Snapshot analysis of representative genomic loci showed that the H3K4me3 signal was nearly absent in MII oocytes, exhibited stage-specific fluctuations during early cleavage stages, and was subsequently established as sharp promoter-proximal peaks throughout preimplantation development, culminating in ESCs where H3K27me3 was also deposited across the corresponding regions (Figure 2F). Together, these results suggest that H3K4me3 at bivalent regions undergoes stage-specific dynamics during preimplantation development.

### 2.3. Dynamics of Chromatin Accessibility at Bivalent Regions During Sheep Early Embryonic Development

**Figure 3:**
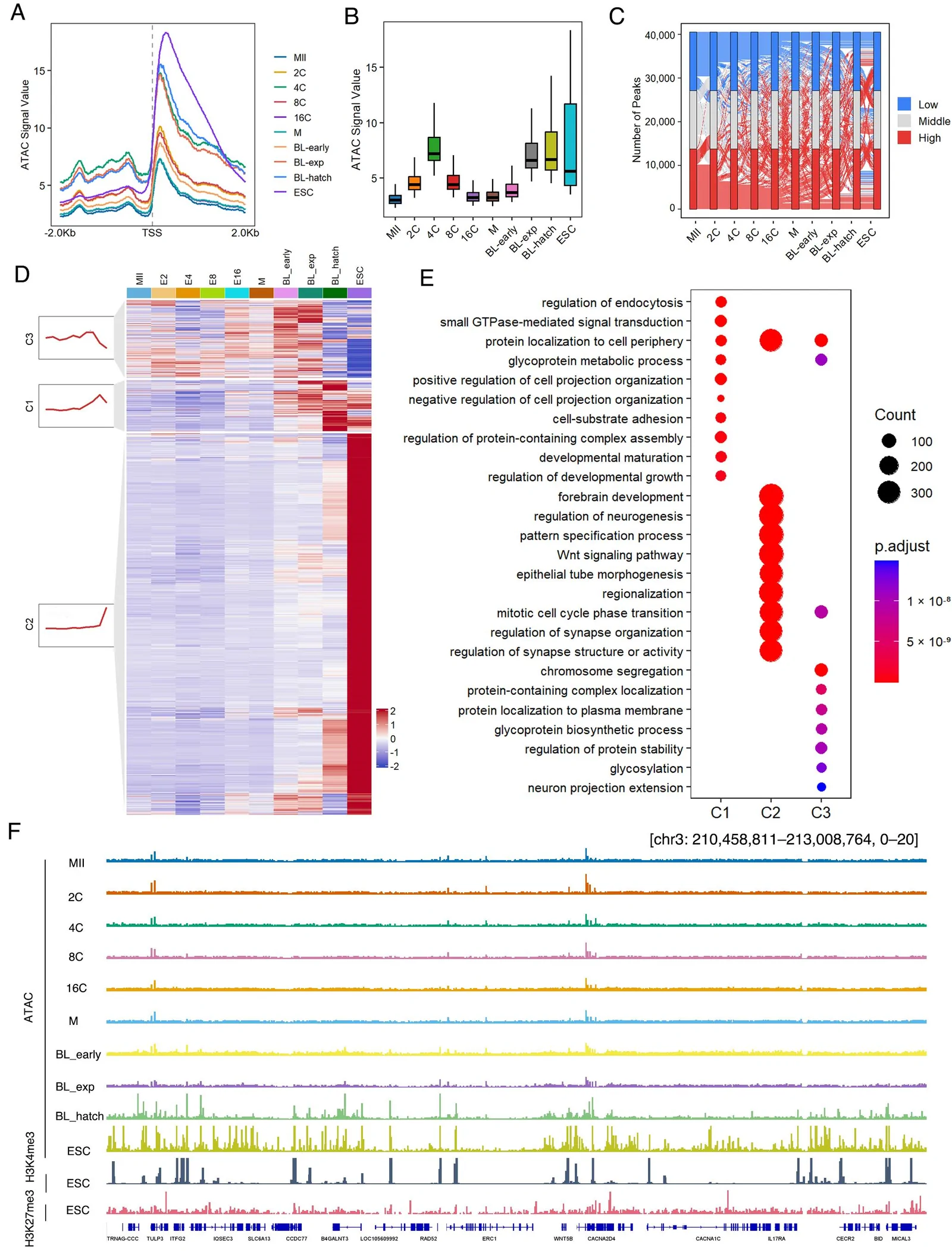
Dynamics of chromatin accessibility at bivalent regions during sheep early embryonic development. (**A**) Metaplot of ATAC mean coverage at TSSs across MII oocytes, 2-, 4-, 8- and 16-cell stage embryos, morula, early blastocysts (BL-early), expanded blastocysts (BL-exp), hatched blastocysts (BL-hatch), and ESCs. (**B**) Boxplot of genome-wide ATAC-seq signal intensity across the indicated developmental stages. (**C**) Stacked plot showing the numbers of ATAC-seq peaks with low, middle, and high signal intensities at each stage. (**D**) Heatmap and k-means clustering (k = 3) of ATAC signals across developmental stages, with cluster sizes indicated. (**E**) Gene Ontology analysis of genes associated with each chromatin accessibility cluster. (**F**) An IGV snapshot showing ATAC distribution in sheep oocytes and the early embryos, together with ATAC, H3K4me3 and H3K27me3 in ESCs, at the chromosome 3 region.

Accessible chromatin was detected at bivalent regions from MII-stage oocytes to hatching blastocysts and ESCs. Metaplot and boxplot analysis revealed marked stage-specific fluctuations in ATAC signal, with the highest accessibility observed at the 4C stage, followed by a decline during later cleavage stages and a subsequent increase toward the blastocyst and ESC stages (Figure 3A,B). The Sankey plot revealed ATAC dynamics in specific regions during early embryonic development (Figure 3C). ATAC signals at bivalent regions were further classified into three clusters by the k-means algorithm (Figure 3D). In cluster 1 (C1, n = 4059), the ATAC signal showed relatively low accessibility during early and mid-preimplantation stages, followed by a marked increase at the blastocyst stages, and was enriched for genes involved in small GTPase-mediated signal transduction and cell–substrate adhesion. In cluster 2 (C2, n = 30,312), ATAC showed relatively low and stable accessibility throughout preimplantation development, followed by a pronounced increase in ESCs, and was enriched for genes involved in protein localization to the cell periphery, the Wnt signaling pathway, and regulation of synapse organization. In cluster 3 (C3, n = 6172), ATAC showed relatively elevated accessibility at early cleavage stages with a further increase toward the blastocyst stages, followed by attenuation in ESCs, and was enriched for genes involved in protein localization and chromosome segregation (Figure 3D,E). Snapshot analysis of representative genomic loci showed that the ATAC signal was relatively low at the MII stage and underwent non-linear, stage-specific remodeling throughout preimplantation development, with a transient peak at early cleavage stages and prominent accessible peaks at the blastocyst and ESC stages, where H3K4me3 and H3K27me3 signals were both enriched at the corresponding regions (Figure 3F). Together, these results suggest that ATAC at bivalent regions undergoes stage-specific dynamics during preimplantation development.

### 2.4. Expression Profiling of Bivalent-Associated Genes During Early Embryonic Development

**Figure 4:**
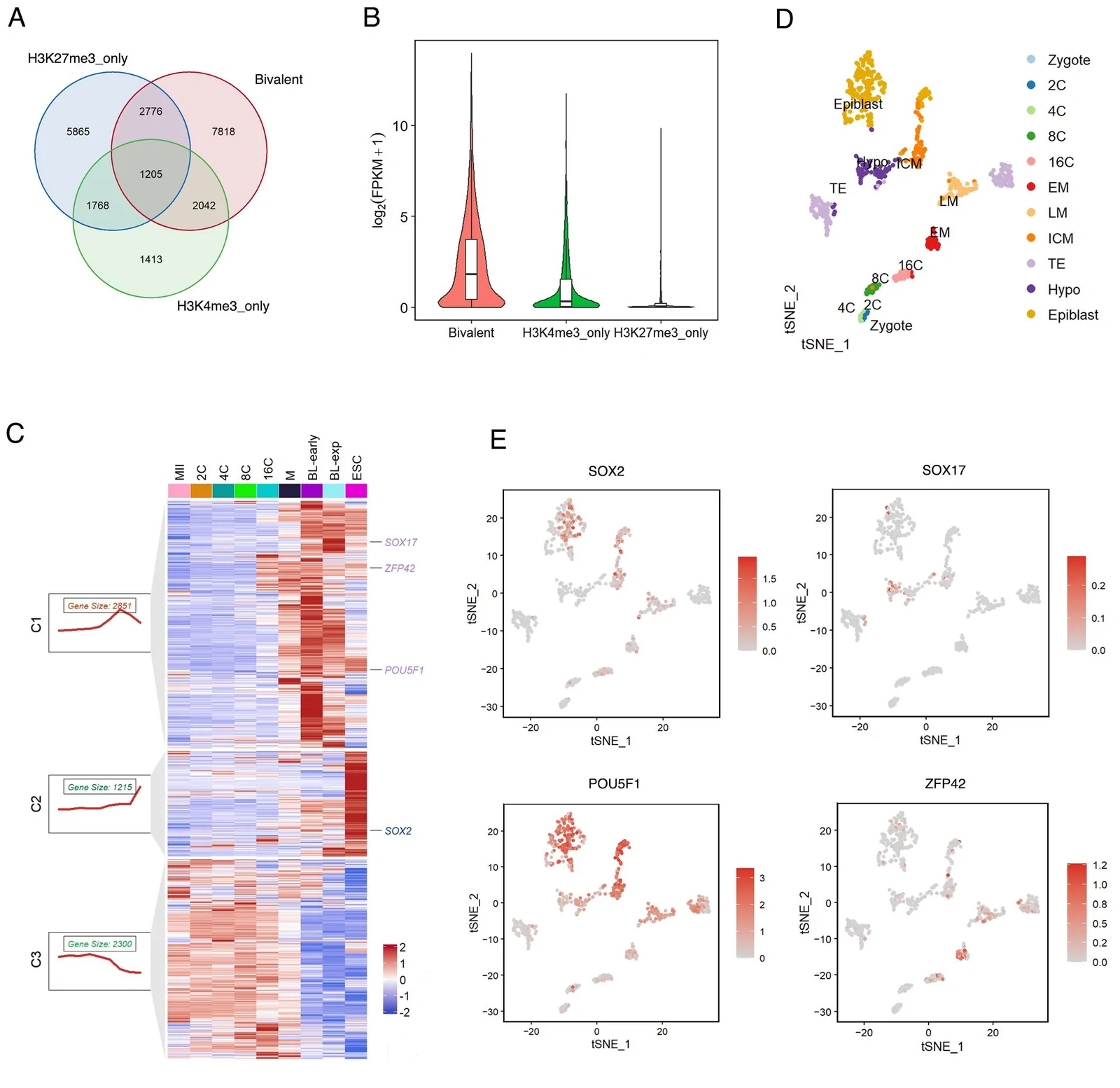
Expression profiling of bivalent-associated genes during early embryonic development. (**A**) The relationship among genes marked by H3K27me3-only, bivalent, and H3K4me3-only modifications. (**B**) Violin plots of expression levels for genes with bivalent, H3K4me3-only, and H3K27me3-only promoter marks. (**C**) Heatmap and k-means clustering (k = 3) of bivalent genes across stages, with representative pluripotency genes indicated. Expression values are Z-score-normalized FPKM (centered per gene across stages), and cluster boundaries correspond to the three k-means groups defined on this Z-scored matrix. (**D**) t-SNE plot of single-cell transcriptomes from zygote to epiblast stages, with major cell lineages annotated. (**E**) Feature t-SNE plots showing the expression of *SOX2*, *SOX17*, *POU5F1*, and *ZFP42* across single-cell lineages.

To characterize the expression features of bivalent-associated genes, we next analyzed bulk and scRNA-seq data from sheep early embryos. As shown in the Venn diagram, H3K27me3-only regions were mapped to 5865 genes, bivalent regions to 7818 genes, and H3K4me3-only regions to 1413 genes (Figure 4A). Because individual genes could be associated with more than one chromatin state, these gene sets were not mutually exclusive. For subsequent gene expression comparison, we therefore used only the mutually exclusive Venn-diagram sectors, comprising 5865 H3K27me3-only genes, 7818 bivalent-only genes, and 1413 H3K4me3-only genes. Expression analysis based on these non-overlapping gene sets showed distinct expression patterns among the three chromatin categories (Figure 4B). k-means clustering of the bulk RNA-seq data stratified bivalent-associated genes into three subgroups with distinct temporal patterns. Genes in cluster 1 (C1, n = 2851) were highly expressed specifically at the blastocyst stage, including *SOX17*, *POU5F1*, and *ZFP42*. Genes in cluster 2 (C2, n = 1215) were expressed exclusively at the ESC stage, including *SOX2*. Genes in cluster 3 (C3, n = 2300) were continually expressed from the MII oocyte to morula stages (Figure 4C). Feature plot analysis of the single-cell RNA-seq (scRNA-seq) data revealed that *SOX2* was specifically highly expressed in pluripotent cell populations including the ICM and epiblast, *SOX17* exhibited specific expression in hypoblast-related cells, *POU5F1* was broadly and highly expressed across pluripotent cell populations, and *ZFP42* was markedly enriched in early cleavage-stage cells (Figure 4D,E), patterns that were largely consistent with the stage-specific expression observed in the bulk RNA-seq. Together, these results indicate that bivalent-associated genes are not uniformly poised but comprise subgroups with distinct, stage-specific transcriptional programs.

### 2.5. Distinct Transcriptional Activity and Chromatin Accessibility at Bivalent Regions Between Formative and Primed ESCs

**Figure 5:**
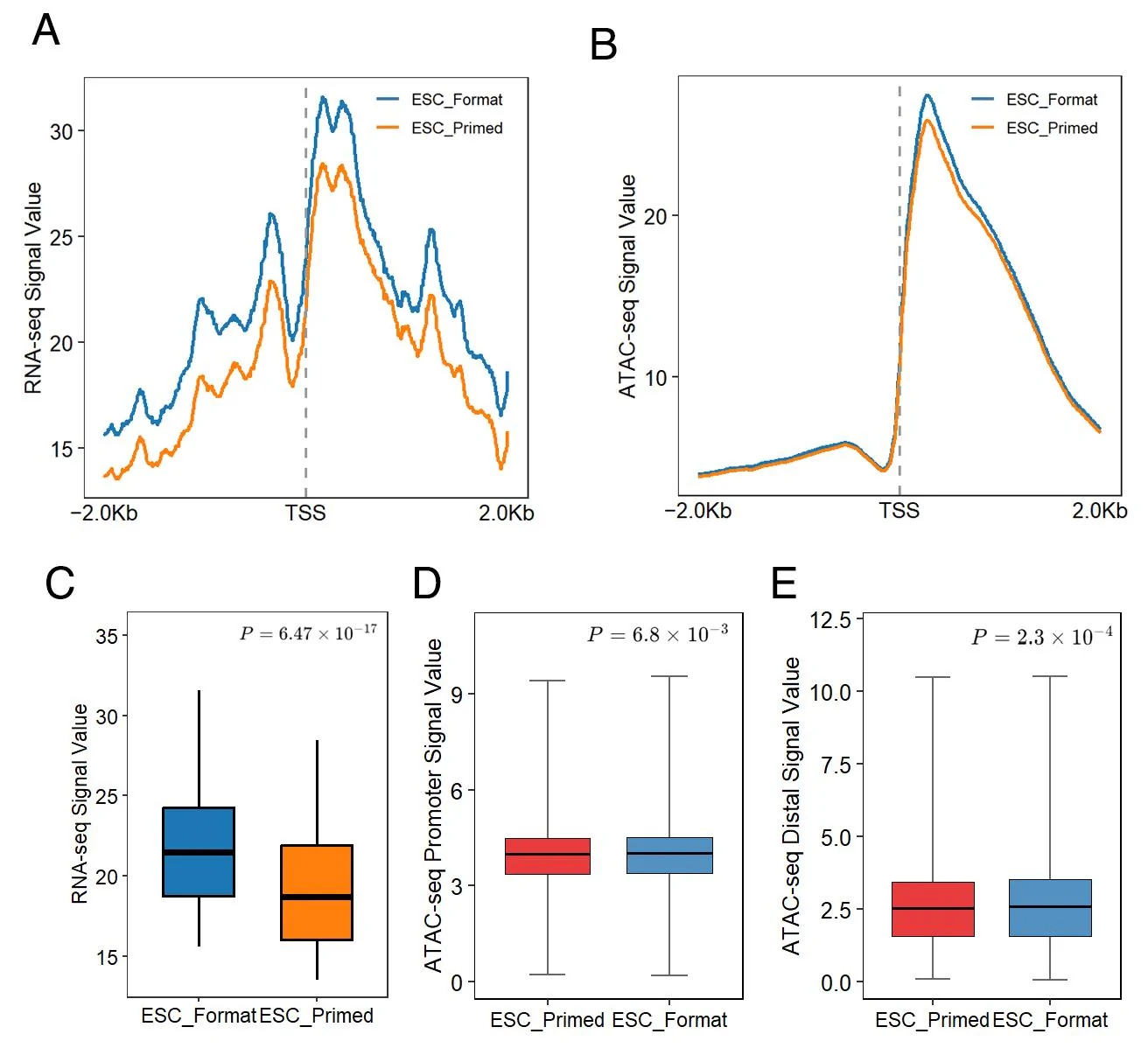
Distinct transcriptional activity and chromatin accessibility at bivalent regions between formative and primed ESCs. (**A**) Metaplot of RNA mean coverage at TSSs in formative and primed ESCs. (**B**) Boxplot of ATAC signal intensity in formative and primed ESCs. *p*-values from a two-sided Wilcoxon test. (**C**) Quantitative comparison of RNA-seq signal intensity between formative and primed ESCs. The effect size was assessed using Cliff’s δ. (**D**) Quantitative comparison of ATAC-seq signal intensity at promoter-proximal bivalent regions in primed (n = 400) and formative (n = 400) ESCs. (**E**) Quantitative comparison of ATAC-seq signal intensity at distal bivalent regions in primed (n = 400) and formative (n = 400) ESCs. *p*-values were calculated using a two-sided Wilcoxon rank-sum test.

To examine whether ESC-defined bivalent regions are associated with distinct regulatory features between pluripotent states, we reanalyzed publicly available RNA-seq and ATAC-seq datasets of formative and primed sheep ESCs generated by Zhang et al. [9]. The reanalyzed RNA-seq data showed higher transcriptional activity around the transcription start sites (TSSs) of bivalent-associated genes in formative ESCs than in primed ESCs (Figure 5A), with a moderate quantitative difference between the two pluripotent states (Cliff’s δ = 0.34, 95% CI: 0.27–0.42; Figure 5C). The reanalyzed ATAC-seq data showed a similar overall pattern, with higher accessibility around the TSSs of bivalent-associated genes in formative ESCs (Figure 5B).

We next examined chromatin accessibility separately at promoter-proximal and distal bivalent regions. Formative ESCs showed a slightly higher ATAC-seq signal than primed ESCs at both promoter-proximal and distal regions (Figure 5D,E). However, the corresponding effect sizes were close to zero for promoter-proximal regions (Cliff’s δ = −0.019, 95% CI: −0.032 to −0.005) and distal regions (Cliff’s δ = −0.019, 95% CI: −0.029 to −0.009), indicating negligible differences in chromatin accessibility between the two pluripotent states. Thus, although the direction of the difference was consistent across promoter-proximal and distal bivalent regions, the magnitude of the difference was small.

Together, these analyses indicate that formative and primed sheep ESCs differ in transcriptional activity around bivalent-associated genes, whereas no meaningful differences in chromatin accessibility exist within promoter-proximal and distal bivalent regions.

## 3. Discussion

Identification and characterization of the genomic distribution and remodeling of bivalent chromatin in sheep ESCs is critical for understanding its mechanisms. To address this limitation, we generated a genome-wide map of bivalent chromatin in sheep ESCs and tracked the coordinated dynamics of H3K4me3, chromatin accessibility, and transcription at these regions across preimplantation development.

ESC-defined bivalent regions undergo dynamic but distinct remodeling of H3K4me3 enrichment and chromatin accessibility during preimplantation development. H3K4me3 is generally enriched around transcription start sites and associated with transcriptionally active promoters of developmental genes [22,29,30,31]. Here, we show that H3K4me3 enrichment at ESC-defined bivalent regions is lowest at the 4C stage among the developmental stages examined and increases progressively toward the blastocyst and ESC stages. Individual regions followed distinct trajectories, indicating that H3K4me3 is dynamically remodeled rather than maintained at a constant level. At early stages, the H3K4me3 signal around TSSs was weaker than at later stages. This is consistent with previous findings that H3K4me3 occupies broad domains in early embryos and becomes restricted to TSSs only later [32]. Thus, the increase in H3K4me3 at bivalent regions is due in part to redistribution of the mark rather than to its net accumulation. In contrast, chromatin accessibility at bivalent regions peaked at the 4C stage, declined during later cleavage stages, and increased again toward the blastocyst and ESC stages [33]. These opposing changes in H3K4me3 and ATAC in bivalent regions at the 4C stage suggest that ESC-defined bivalent regions undergo stage-specific, distinct chromatin remodeling during preimplantation development, prompting further examination of their transcriptional dynamics.

Comparable developmental remodeling of chromatin accessibility and histone modifications has also been reported in other mammalian species. In bovine embryos, chromatin accessibility undergoes substantial remodeling during preimplantation development, with pronounced changes around the period of embryonic genome activation [34], and changes in accessibility can precede the establishment of H3K4me3 at some regulatory regions [35]. Comparative studies of mouse, human, and bovine embryos further indicate that chromatin remodeling during early development shares some conserved features, although the timing of embryonic genome activation and the associated regulatory dynamics differ among species [36]. Thus, the H3K4me3 and chromatin accessibility dynamics observed in sheep are broadly consistent with developmental chromatin remodeling reported in other mammals, rather than providing evidence for a sheep-specific pattern. However, the precise timing and magnitude of these changes may reflect species-specific developmental programs.

The developmental remodeling of bivalent regions was accompanied by marked changes in the transcriptional states of their associated genes. Bivalent domains have traditionally been proposed to maintain developmental regulators in a poised state for subsequent activation or repression during differentiation [20]. Alternatively, bivalency has been suggested to be associated with CpG-rich developmental promoters and protection from aberrant DNA methylation. However, in our data, bivalent-associated genes showed the highest expression among the three chromatin categories within the developmental-regulatory subset. Their expression also varied across developmental stages, with k-means clustering identifying three clusters showing preferential expression at the ESC stage, the blastocyst stage and the MII-to-morula interval [37], respectively. Moreover, representative transcription factors, including POU5F1, SOX2, SOX17, and ZFP42, displayed stage-dependent expression patterns broadly consistent with their established roles in pluripotency and lineage specification [9]. These findings indicate that bivalent-associated genes exhibit heterogeneous and developmentally regulated transcriptional states rather than a uniform transcriptional pattern.

The stage-dependent variation observed above further prompted us to examine whether the chromatin and transcriptional states of bivalent regions also differ between distinct pluripotent states. Formative and primed ESCs represent distinct states along the pluripotency continuum and are characterized by different regulatory landscapes [38,39,40,41]. In the reanalyzed public datasets of formative and primed sheep ESCs [9], formative ESCs showed higher transcriptional activity around the TSSs of bivalent-associated genes than primed ESCs. The corresponding ATAC-seq data showed a similar overall pattern around TSSs, whereas accessibility differences within promoter-proximal and distal bivalent regions themselves were small. These findings are consistent with previous evidence that chromatin accessibility and bivalent chromatin features can vary across pluripotent states [38,39,40,41], but do not by themselves establish a functional role for bivalency in maintaining a particular pluripotent state. Thus, the chromatin and transcriptional states of bivalent regions are associated with the pluripotency state rather than being invariant features of sheep ESCs. Together with their developmental remodeling during preimplantation development, these findings support the view that bivalent regions represent a dynamic chromatin state that varies across both developmental stages and pluripotent states.

Beyond its research value, the present dataset may also have practical implications for sheep ESC biology and production. A better understanding of how bivalent chromatin states are established and regulated in sheep ESCs provides a rational basis for improving ESC derivation and culture: the H3K4me3/H3K27me3 state at developmental genes can serve as an epigenetic readout of ESC quality and of pluripotency-state stability during long-term culture, expansion, and genetic engineering. Because stable, well-characterized sheep ESCs are a prerequisite for stem-cell-based breeding programs, including ESC-mediated genome editing and dissemination of favorable alleles, epigenetic monitoring of the bivalent landscape may directly support the scalable application of these technologies in an economically important ruminant. More broadly, the stage-specific H3K4me3 enrichment and chromatin accessibility dynamics identified at bivalent regions provide candidate molecular features for future studies of embryo developmental competence in in vitro embryo production systems that underpin embryo transfer and MOET programs, complementing conventional morphological grading, although their practical value as indicators requires further validation.

Future studies should focus on experimentally testing the regulatory relationships suggested by the present multi-omics analyses. Gain- or loss-of-function experiments targeting candidate transcription factors, together with targeted analyses of histone modifications, chromatin accessibility, and gene expression, could help determine their causal roles in bivalent chromatin regulation. Integrating additional epigenomic features, such as H3K27ac, H3K4me1, DNA methylation, and RNA polymerase II occupancy, as well as cell-resolved datasets, may further clarify how bivalent regions are established and remodeled during embryonic development and lineage specification.

Several limitations should be acknowledged in the present study. The multi-omics maps generated here provide a descriptive framework for bivalent chromatin organization in sheep ESCs and preimplantation embryos, but they do not directly establish the causal mechanisms underlying the establishment, maintenance, and resolution of bivalent domains. In particular, how transcription factors and chromatin-modifying complexes cooperate to regulate H3K4me3 and H3K27me3 at developmentally regulated promoters, and how these domains are resolved during lineage commitment, remain to be experimentally determined. In addition, the relationship between bivalent chromatin and other regulatory layers, including DNA methylation, enhancer activity, and higher-order chromatin organization, was not comprehensively addressed in the present study. Future studies combining targeted perturbation of candidate regulators with time-resolved profiling during ESC differentiation and early lineage commitment will therefore be important for distinguishing causal regulatory relationships from correlations and for developing a more integrated model of bivalent chromatin regulation during sheep early embryonic development.

## 4. Materials and Methods

### 4.1. Sheep ESC Culture

The sheep ESC line used for CUT&Tag and ATAC-seq profiling was established in our laboratory, and detailed characterization data will be presented elsewhere. Briefly, sheep blastocysts were treated with pronase for 5 min to remove the zona pellucida, and the inner cell mass was isolated and seeded onto mitomycin C-inactivated mouse embryonic fibroblast (MEF) feeder layers. Cells were cultured following the bovine expanded potential stem cell culture system. After colony formation, cells were continuously passaged. When the ESC colonies reached approximately 80% confluence, cells were digested with TrypLE and passaged at a ratio of 1:10–1:12 onto new MEF feeder layers for expansion. The three biological replicates used for CUT&Tag, ATAC-seq, and RNA-seq profiling were independent culture batches (passages) of this single ESC line, rather than independently derived ESC lines.

### 4.2. Collection of Sheep Early Embryos

Ovaries from Hu sheep were collected at a local abattoir and transported to the laboratory in warm saline within 2–4 h. Cumulus–oocyte complexes (COCs) were aspirated from antral follicles (2–6 mm), selected for compact cumulus and uniform ooplasm, and matured in vitro in TCM-199-based medium for 22–24 h at 38.5 °C and 5% CO_2_. Matured oocytes were co-incubated with swim-up-capacitated Hu sheep semen in IVF medium (1 × 10^6^ motile sperm/mL), and the time of sperm addition was defined as 0 h post-insemination (hpi). Presumptive zygotes were denuded at 18–20 hpi and cultured in SOF + 0.8% BSA at 38.5 °C under 5% CO_2_, 5% O_2_, and 90% N_2_ throughout early embryonic development. Embryos at different developmental stages were collected directly from in vitro culture at the following time points: MII oocytes were harvested immediately after IVM (i.e., before IVF); 2-cell embryos at approximately 24–30 hpi, 4-cell embryos at 36–42 hpi, 8-cell embryos at 48–54 hpi, 16-cell embryos at 60–66 hpi, and morula (M) at approximately 96 hpi were sampled from culture droplets; blastocysts (BLs) at approximately 144–168 hpi were further classified into early blastocysts (BL-early), expanded blastocysts (BL-expanded, BL-exp), and hatching blastocysts (BL-hatching, BL-hatch) based on morphology. For CUT&Tag, approximately 50–100 embryos were collected per biological replicate at each developmental stage; for ATAC-seq, approximately 200–500 embryos were collected per biological replicate; and for RNA-seq, 5 embryos were collected per biological replicate. Three independent biological replicates were collected for each developmental stage, with each obtained from an independent batch of in vitro embryo production.

### 4.3. CUT&Tag Library Construction and Sequencing

CUT&Tag libraries were constructed using the NovoNGS CUT&Tag 4.0 High-Sensitivity Illumina Kit (Cat# N259-YH01, Novogene, Nanjing, China) following the manufacturer’s instructions [42,43]. Briefly, embryo samples were treated with acidic Tyrode’s solution (Cat# T1788, Sigma-Aldrich, St. Louis, MO, USA) to remove the zona pellucida and washed with washing buffer. ESC samples were digested with Accutase into single cells and used directly. Primary antibodies, rabbit anti-H3K4me3 (Cat# ab213224, 1:100 dilution, Abcam, UK) or rabbit anti-H3K27me3 (Cat# ab192985, 1:100 dilution, Abcam, Cambridge, UK), were added and incubated overnight at 4 °C (Table S2). Goat anti-rabbit secondary antibody (Cat# N269-01A, Novogene) was added and incubated at room temperature for 1 h. After three washes with washing buffer, pAG-transposase complex containing 1% digitonin was added and incubated at room temperature for 1 h, followed by transfer to the fragmentation reaction system and incubation at 37 °C for 1 h to complete DNA fragmentation. DNA was recovered using fragmentation DNA extraction magnetic beads, and PCR amplification was performed with i5 and i7 sequencing primers (15 cycles). Each library was constructed from an independent biological replicate as described above. Qualified libraries were submitted to Beijing Annoroad Gene Technology Co. (Beijing, China) for high-throughput sequencing on the Illumina NovaSeq 6000 platform (PE150, llumina, Inc., San Diego, CA, USA).

### 4.4. ATAC-Seq Library Construction and Sequencing

ATAC-seq was performed following the Omni-ATAC protocol described by Corces et al. [44]. For ESC samples, approximately 5 × 10^4^ cells were collected, washed with cold ATAC-Resuspension Buffer (RSB: 10 mmol/L Tris-HCl pH 7.4, 10 mmol/L NaCl, 3 mmol/L MgCl_2_), and lysed in cold lysis buffer containing 0.1% Tween-20, 0.1% NP-40, and 0.01% Digitonin on ice for 3 min. Nuclei were collected by centrifugation. For early embryo samples (MII, 2C, 4C, 8C, 16C, M, BL-early, BL-exp, BL-hatch), approximately 200–500 embryos per batch were used to prepare nuclei in lysis buffer containing 0.1% Tween-20 and 0.01% Digitonin. The Tn5 transposase reaction system was added and incubated at 37 °C for 30 min for in situ transposition. After termination of the reaction, PCR amplification was performed (initial 5 cycles, followed by 3–7 additional cycles based on qPCR monitoring). Libraries were purified using AMPure XP beads (Beckman Coulter, Brea, CA, USA) with double-sided size selection (0.5×–1.5×). Qualified libraries were sequenced on the Illumina NovaSeq 6000 platform (PE150).

### 4.5. CUT&Tag Data Analysis

Paired-end sequencing reads were aligned to the sheep reference genome oviAri4 (Oar_v4.0; NCBI assembly accession GCF_000298735.2) using Bowtie2 (v2.5.4). Gene annotations were obtained from NCBI Annotation Release 102 (12 August 2020), retrieved from the UCSC ncbiRefSeq track for the oviAri4 assembly (snapshot dated 10 February 2021). The aligned reads were sorted and indexed using SAMtools. H3K4me3 and H3K27me3 peaks were called independently for each biological replicate using MACS2 (v2.2.7): H3K4me3 peaks were identified in narrow mode (--nomodel --shift -100 --extsize 200 -p 0.05), whereas H3K27me3 peaks were identified in broad mode (--broad --broad-cutoff 0.05). For each histone modification, genomic intervals supported by all three biological replicates were identified by intersecting the independently called peak sets, followed by merging overlapping intervals to generate a three-replicate consensus peak set for each developmental stage. Library quality was assessed using the fraction of reads in peaks (*FRiP*). For the ESC stage, the FRiP values of the three biological replicates were 0.7091, 0.7272, and 0.7101 for H3K4me3 and 0.8591, 0.5092, and 0.4955 for H3K27me3 (Table S1), indicating that a substantial fraction of mapped reads was associated with the corresponding consensus peak sets. Normalized signal tracks were generated using deepTools (v3.5.6) for genome browser visualization and downstream signal profiling.

### 4.6. ATAC-Seq Data Analysis

Paired-end sequencing reads were aligned to the sheep reference genome oviAri4 using Bowtie2 (v2.5.4). The aligned reads were sorted and indexed using SAMtools, and biological replicates from each dataset were analyzed independently for peak calling. Consensus peak sets were subsequently generated based on the reproducibility of the biological replicates before downstream analyses. Peak calling was performed using MACS2 with parameters optimized for ATAC-seq data. RPKM-normalized signal tracks were generated using deepTools (v3.5.6) for genome browser visualization and downstream signal profiling. Publicly available ATAC-seq data of sheep ESCs representing formative and primed states generated by Zhang et al. [9] were downloaded from the public database and reanalyzed using the same analytical pipeline described above. The bivalent chromatin regions identified in ESCs were used as a common genomic reference to compare chromatin accessibility between formative and primed ESC states. ATAC-seq signal enrichment around these regions and TSSs was profiled using deepTools computeMatrix to characterize accessibility dynamics associated with bivalent chromatin.

### 4.7. RNA-Seq Data Analysis

Reads were trimmed to remove adaptors and low-quality bases using fastp (v1.0.1) and reads that passed quality control were mapped to the reference genome oviAri4 using HISAT2 (v2.2.1). All the mapped reads were then sorted and indexed using SAMtools (v1.22). Transcript quantification was performed through featureCounts (v2.1.1), and the expression of genes was normalized with fragments per kilobase of exon model per million mapped fragments (FPKM). For stage comparisons, expression is presented as the mean ± SEM across the three biological replicates. Publicly available RNA-seq data of sheep ESCs representing formative and primed states generated by Zhang et al. [9] were downloaded from the public database and reanalyzed using the same analytical pipeline described above.

### 4.8. Single-Cell Transcriptome Data Analysis

Publicly available scRNA-seq data of sheep preimplantation embryos generated by Zhang et al. [9] were used to further examine the expression patterns of bivalent-associated genes at the single-cell level. The processed expression data and cell-type annotations were obtained from the original study [9]. Cell populations representing different developmental stages and lineages, including cleavage-stage embryos, morula, inner cell mass (ICM), epiblast, trophectoderm (TE), and hypoblast, were analyzed according to the annotations reported in the original study. The expression patterns of selected bivalent-associated genes and key transcription factors, including SOX2, SOX17, POU5F1, and ZFP42, were examined across the annotated cell populations.

### 4.9. Identification of Bivalent Chromatin Regions

Bivalent chromatin regions were identified based on the genomic overlap of H3K4me3 and H3K27me3 CUT&Tag peaks in sheep ESCs. Genomic regions were categorized as H3K4me3-only, H3K27me3-only, or bivalent according to their overlap with the two histone modification peak sets. Peak intersections were determined using bedtools intersect, and regions with more than 200 bp of overlap between H3K4me3 and H3K27me3 peaks were defined as bivalent regions [45].

### 4.10. Functional Enrichment Analysis

Gene Ontology (GO) over-representation analysis (ORA) of the fixed gene sets defined by peak overlap—bivalent-associated genes (Figure 1F) and the genes associated with each H3K4me3 and ATAC cluster (Figure 2E and Figure 3E)—was performed using clusterProfiler (v4.16.0). Data were analyzed and visualized using R.

### 4.11. Statistical Analysis and Data Visualization

All statistical analyses were performed using R software (v4.5.1). Data are presented as mean ± SEM. Heatmaps and boxplots of volcano plots, gene expression, GO, KEGG, and signal intensity were generated using the R packages pheatmap (v1.0.12) and ggplot2 (v3.3.2), respectively. Two-sided Wilcoxon rank-sum tests were used for pairwise comparisons; because region- and gene-level comparisons involve very large sample sizes, effect sizes (Cliff’s delta) are reported alongside *p*-values for all boxplot comparisons (Figure 1E and Figure 5B–E), and the number of regions or genes per group (n) is indicated in the corresponding figure legends.

## 5. Conclusions

Collectively, our findings show that ESC-defined bivalent regions undergo dynamic, stage-specific changes in H3K4me3 enrichment, chromatin accessibility, and transcription during preimplantation development. The differences observed between formative and primed ESCs further suggest that the chromatin and transcriptional states of these regions vary with the pluripotent state rather than remaining constant. Together, these findings provide evidence that bivalent regions in sheep ESCs represent a dynamic chromatin state associated with developmental and pluripotency transitions and provide a basis for further investigating their potential roles in early embryonic development.

## Data Availability

The original contributions presented in this study are included in the article/Supplementary Materials. Further inquiries can be directed to the corresponding author.

## Supplementary Materials

The following supporting information can be downloaded at https://www.mdpi.com/article/10.3390/ijms27209192/s1.
